# Bilateral and multiple cavitation sounds during upper cervical thrust manipulation

**DOI:** 10.1186/1471-2474-14-24

**Published:** 2013-01-15

**Authors:** James Dunning, Firas Mourad, Marco Barbero, Diego Leoni, Corrado Cescon, Raymond Butts

**Affiliations:** 1 Alabama Physical Therapy & Acupuncture, Montgomery, AL, USA; 2 Nova Southeastern University, Fort Lauderdale, FL, USA; 3 Sportlife Physiotherapy, Montichiari, Italy; 4 Department of Health Sciences, University of Applied Sciences and Arts of Southern Switzerland (SUPSI), Manno, Switzerland; 5 University of South Carolina, Columbia, SC, USA

**Keywords:** Cavitation, Popping sound, High velocity thrust manipulation, Upper cervical

## Abstract

**Background:**

The popping produced during high-velocity, low-amplitude (HVLA) thrust manipulation is a common sound; however to our knowledge, no study has previously investigated the location of cavitation sounds during manipulation of the upper cervical spine. The primary purpose was to determine which side of the spine cavitates during C1-2 rotatory HVLA thrust manipulation. Secondary aims were to calculate the average number of pops, the duration of upper cervical thrust manipulation, and the duration of a single cavitation.

**Methods:**

Nineteen asymptomatic participants received two upper cervical thrust manipulations targeting the right and left C1-2 articulation, respectively. Skin mounted microphones were secured bilaterally over the transverse process of C1, and sound wave signals were recorded. Identification of the side, duration, and number of popping sounds were determined by simultaneous analysis of spectrograms with audio feedback using custom software developed in Matlab.

**Results:**

Bilateral popping sounds were detected in 34 (91.9%) of 37 manipulations while unilateral popping sounds were detected in just 3 (8.1%) manipulations; that is, cavitation was significantly (P < 0.001) more likely to occur bilaterally than unilaterally. Of the 132 total cavitations, 72 occurred ipsilateral and 60 occurred contralateral to the targeted C1-2 articulation. In other words, cavitation was no more likely to occur on the ipsilateral than the contralateral side (P = 0.294). The mean number of pops per C1-2 rotatory HVLA thrust manipulation was 3.57 (95% CI: 3.19, 3.94) and the mean number of pops per subject following both right and left C1-2 thrust manipulations was 6.95 (95% CI: 6.11, 7.79). The mean duration of a single audible pop was 5.66 ms (95% CI: 5.36, 5.96) and the mean duration of a single manipulation was 96.95 ms (95% CI: 57.20, 136.71).

**Conclusions:**

Cavitation was significantly more likely to occur bilaterally than unilaterally during upper cervical HVLA thrust manipulation. Most subjects produced 3–4 pops during a single rotatory HVLA thrust manipulation targeting the right or left C1-2 articulation; therefore, practitioners of spinal manipulative therapy should expect multiple popping sounds when performing upper cervical thrust manipulation to the atlanto-axial joint. Furthermore, the traditional manual therapy approach of targeting a single ipsilateral or contralateral facet joint in the upper cervical spine may not be realistic.

## Background

The cracking, popping or clicking noise produced during spinal manipulation is a common sound to physiotherapists, osteopaths and chiropractors [1-9]. Anecdotal evidence and recent studies suggest it is common for a single spinal high-velocity low-amplitude (HVLA) thrust manipulation to produce 2 or more distinctive joint popping sounds [1,4,8-10]. However, the question of whether these multiple popping sounds emanate from the same joint, adjacent ipsilateral or contralateral joints, or even extra-articular soft-tissues has yet to be answered [1,2,4,9,11]. Furthermore, to our knowledge only two studies [2,8] have previously investigated this phenomenon in the cervical spine.

While the exact mechanism and origin of the popping sound during HVLA thrust manipulation remains relatively unknown, [11] the predominant theory is still the cavitation model of joint cracking originally proposed by Unsworth in 1971 [12]. That is, radiolucent cavities or intra-articular gas bubbles have been observed on plain film radiographs following distractive thrust manipulations of the third metacarpophalangeal (MCP) joints. Furthermore, an increase in the joint space and a decrease in joint density have also been demonstrated in the MCP joints post-manipulation [12-14]. In theory, a rapid increase in the joint volume occurs during manual manipulation of the MCP joint, subsequently dropping the partial pressure of carbon dioxide within the synovial fluid and allowing it to be released as a gaseous bubble into the joint cavity [7,12,14-18]. The subsequent flow of synovial fluid into the low pressure regions of the cavity collapses the gas bubbles, producing the audible cracking sound [13,17].

Although the source of the cracking sound in the MCP joints has typically been associated with the cavitation phenomenon, [12,17] Cascioli et al. [11] found no evidence of gas in the zygapophyseal joint space on CT scans and plain film images immediately following both traction and traction-free lower cervical HVLA thrust manipulations. That is, no significant change in the width, area or density values of the cervical zygapophyseal joint spaces were found immediately after lower cervical HVLA thrust manipulation [11]. Therefore, it is still unknown if the cavitation phenomenon takes place in spinal facet joints, because to date, the cavitation or vacuum phenomenon has never actually been visualized or recorded in articulations of the cervical spine during or following thrust manipulation [11].

The cavitation sound is traditionally considered to be an important indicator for the successful technical delivery of an HVLA thrust manipulation; [5,7,14,19-21] however, two studies [22,23] by a single research team found the audible pop following thrust manipulation to the lumbopelvic region may not relate to improved outcomes in patients with nonradicular low back pain. Nevertheless, the production of popping sounds is anecdotally still believed by many practitioners to be an indicator of the effectiveness of a joint manipulative treatment [3,6,15,24] and may explain why researchers frequently repeat the HVLA thrust manipulation if they did not hear or palpate popping sounds on the first attempt [5,19-21,25].

To our knowledge, there are only two previous studies [2,8] that have investigated the side of joint cavitation associated with cervical spine manipulation, and neither of them involved the upper cervical spine. During “lateral to medial and rotatory” HVLA thrust manipulation targeting the C3-4 facet joint, Reggars and Pollard [8] found 47 (94%) of the 50 subjects exhibited cracking sounds on the contralateral side to the applicator contact, while 2 subjects exhibited bilateral sounds and one subject an ipsilateral sound. The second and most recent study to investigate the side of joint cavitation associated with cervical spine manipulation was done by Bolton et al [2]. Following C3-4 thrust manipulations in 20 asymptomatic subjects, Bolton et al. [2] found the popping was significantly more likely to occur on the contralateral side to the applicator for rotation thrusts. In contrast, thrusts with a primary lever of side-bending resulted in audible pops that were no more likely to occur on the ipsilateral than the contralateral side of the applicator.

The expectation of one single pop emanating from the target or dysfunctional facet joint during HVLA thrust manipulation is therefore not consistent with the existing literature for the lower cervical, [8,10] thoracic [9] or lumbar [1,4,9] regions. Moreover, both anecdotal evidence and the existing literature suggest that it is common for one HVLA thrust manipulation to produce 2 or more distinctive joint popping sounds [1,8-10].

Reggars & Pollard [8] reported only 36% of the cervical thrust manipulations targeting the C3-4 articulation produced a single audible cavitation and that up to 4–5 cavitations were evident in some subjects. Likewise, using time and amplitude analysis of digitally recorded sound signals following rotatory thrust manipulations directed to the C3-4 zygapophyseal joints in 50 asymptomatic subjects, Reggars [10] reported that 64% (32/50) of participants produced 2 or 3 distinct joint crack signals, 18% (9/50) produced a single audible pop, 14% (7/50) produced four pops, and 4% (2/50) produced five separate crack signals. In total, 50 manipulations on the 50 subjects produced 123 individual joint cracks, resulting in a mean of 2.46 pops per manipulation. Likewise, using accelerometers taped to the skin over the spinal column, Ross et al. [9] found most thoracic and lumbar thrusts produced 2–6 audible popping sounds with an average error from the target joint of 3.5 cm and 5.29 cm, respectively.

By analyzing force-time history graphs, Triano [26] measured the duration of the thrusting procedure for a C2-3 lateral break manipulation to be 135 ms. Likewise, for lower cervical manipulations, Ngan et al. [27] and Herzog et al. [7,28] reported a mean thrust duration of 158 ms and 80–200 ms, respectively. However, the duration of upper cervical HVLA thrust manipulation has yet to be investigated by any study.

Although Herzog et al. [7] used a mechanical accelerometer during T4 posterior to anterior thrust manipulations in 28 patients with thoracic spine pain and reported triphasic “cavitation signals” with a mean duration of 20 ms; whether this value represents a single popping sound or multiple popping sounds remains unclear. That is, although “cavitation signals” were indirectly measured by Herzog et al., [7] they did not directly measure any sound wave signals to calculate the duration of an individual pop. Using sound wave recordings and following thrust manipulation of the metacarpophalangeal joints, Sandoz et al. [14] and Meal and Scott [29] reported “cavitation signals” with mean durations of 40–60 ms and 25–75 ms, respectively; however to date, no study has measured the value associated with 95% of the instantaneous energy burst and used it to calculate the duration of the popping sounds that occur during spinal manipulation [7,14,29].

To date and to our knowledge, no study has investigated the side, duration or number of audible popping sounds during upper cervical HVLA thrust manipulation. Therefore, the primary purpose of the study was to determine which side of the upper cervical spine cavitates during rotatory C1-2 HVLA thrust manipulation. Secondary aims of the study were to calculate the duration of a single cavitation or popping sound, the duration of a single upper cervical thrust manipulation procedure, and the average number of popping sounds following C1-2 HVLA thrust manipulation.

## Methods

### Participants

Nineteen asymptomatic subjects (10 females and 9 males) were recruited by convenience sampling former patients from a private physical therapy outpatient clinic in Brescia, Italy during July of 2011. Their ages ranged between 18 and 52 years with a mean (SD) of 26.4 (8.6) years. Height ranged between 161 and 183 cm with a mean (SD) of 172.0 (7.3) cm. Weight was 46.0 kg to 110.0 kg with a mean (SD) of 68.3 (15.6) kg.

Before any experimental procedures, all subjects completed a medical history questionnaire and underwent a physical examination intended to screen for relative and absolute contraindications for cervical manipulation. For subjects to be eligible, they had to present with no neck pain (defined as pain in the region between the superior nuchal line and first thoracic spinous process) over the past 3 months and be between 18 and 70 years of age. This study was approved by the Corporate Clinical Research Ethics Committee and all subjects provided informed consent before their participation in the study.

Patients were excluded if they exhibited any red flags (i.e., tumor, fracture, metabolic diseases, rheumatoid arthritis, osteoporosis, resting blood pressure greater than 140/90 mmHg, prolonged history of steroid use, etc.), presented with 2 or more positive neurologic signs consistent with nerve root compression (muscle weakness involving a major muscle group of the upper extremity, diminished upper extremity deep tendon reflex, or diminished or absent sensation to pinprick in any upper extremity dermatome), presented with a diagnosis of cervical spinal stenosis, exhibited bilateral upper extremity symptoms, had evidence of central nervous system disease (hyperreflexia, sensory disturbances in the hand, intrinsic muscle wasting of the hands, unsteadiness during walking, nystagmus, loss of visual acuity, impaired sensation of the face, altered taste, the presence of pathological reflexes), had a history of whiplash injury within the previous 3 months, had prior surgery to the neck or thoracic spine, or had neck pain within the previous 3 month period. Of the 20 asymptomatic, former patients that were invited to enter the study, none refused participation; however, one subject was excluded due to a history of a recent injury.

The most recent literature suggests that pre-manipulative cervical artery testing is unable to identify those individuals at risk of vascular complications from cervical HVLA thrust manipulation, [30,31] and any symptoms detected during pre-manipulative testing may be unrelated to changes in blood flow in the vertebral artery. Therefore, a negative test neither predicts the absence of arterial pathology nor the propensity of the artery to be injured during cervical HVLA thrust manipulation, with testing neither sensitive or specific [30-34]. Screening questions for cervical artery disease were negative, and pre-manipulative cervical artery testing was not used.

### Manipulative physiotherapist

A single, U.S. licensed physical therapist performed all of the upper cervical HVLA thrust manipulations in this study. At the time of data collection, the physical therapist had completed a post-graduate Master of Science in Advanced Manipulative Therapy, had worked in clinical practice for 12 years, and routinely used upper cervical HVLA thrust manipulation in daily practice.

### C1-2 rotatory HVLA thrust manipulation technique

**Figure 1:**
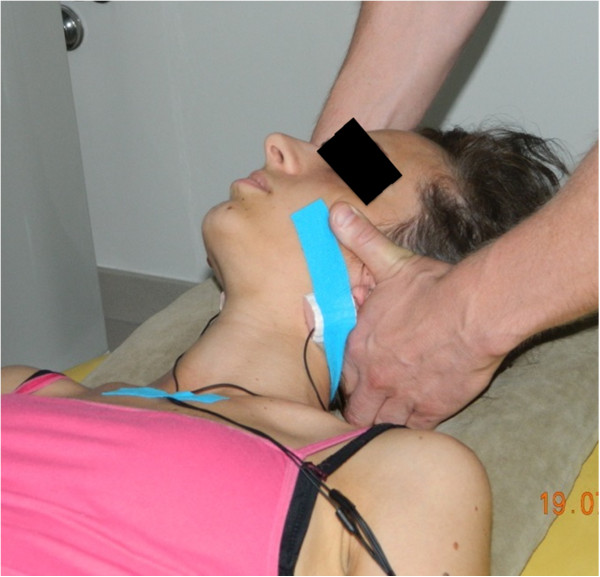
High-velocity low-amplitude thrust manipulation directed to the left C1-2 articulation.

A single rotatory HVLA thrust manipulation directed to the upper cervical spine (C1-2) with the patient supine was performed (Figure 1). For this technique, [5,6,35] the patient’s right posterior arch of the atlas was contacted with the lateral aspect of the proximal phalanx of the therapist’s right second finger using a “cradle hold”. To localize the forces to the right C1-2 articulation, secondary levers of extension, posterior-anterior translation, right (ipsilateral) lateral-flexion and left (contralateral) lateral translation were used [5,6,35]. While maintaining the secondary levers, the therapist performed a single HVLA thrust manipulation to the right atlanto-axial joint using the combined primary thrusting levers of left rotation in an arc toward the underside eye and translation toward the table [5,6,35]. This was repeated using the same procedure but directed to the left C1-2 articulation. Prior to data collection, the target side and delivery order of the C1-2 rotatory HVLA thrust manipulations were randomized using a table of randomly assigned numbers for all subjects. Popping or cracking sounds were heard on all HVLA thrust manipulations; hence, there was no need for second attempts.

### Microphone placement and sound wave collection

**Figure 2:**
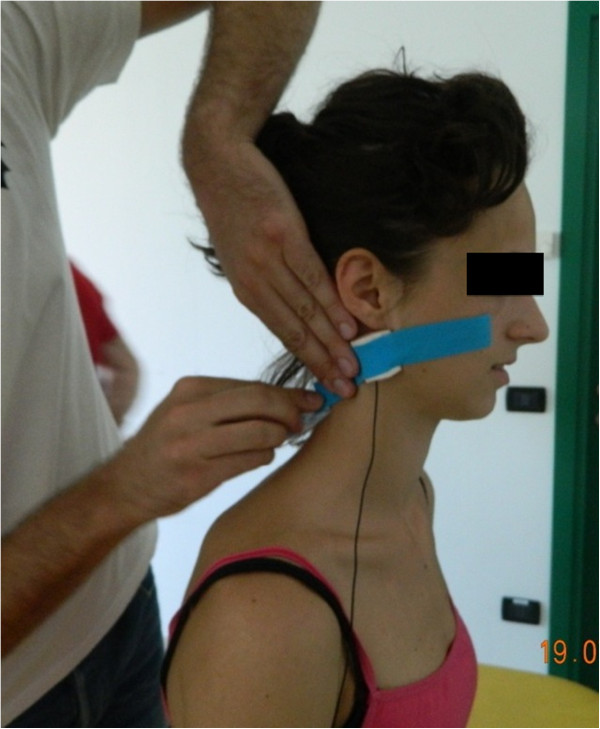
Placement and securing of skin mounted microphone over the lateral aspect of the transverse process of the atlas.

After physical examination and prior to the delivery of upper cervical HVLA thrust manipulation, skin mounted microphones were secured bilaterally over the lateral aspect of the transverse process of C1 (Figure 2). Each microphone was labeled with a right and left tag. The microphones were connected to a data acquisition system (MOTU 8pre 16 bit, Cambridge, MA) and a MacBook Pro laptop with custom developed software for audio acquisition. With two channels in place, microphones were then checked for sound detection to ensure they were online and recording the correct side. Sampling frequency was set at 44,100 Hz. With the order randomized, all subjects then received two HVLA thrust manipulations: one targeting the left C1-2 joint, and one targeting the right C1-2 joint. The sound wave signals and resultant popping sounds during the upper cervical HVLA thrust manipulations were recorded for later data extraction and analysis.

### Data analysis

**Figure 3:**
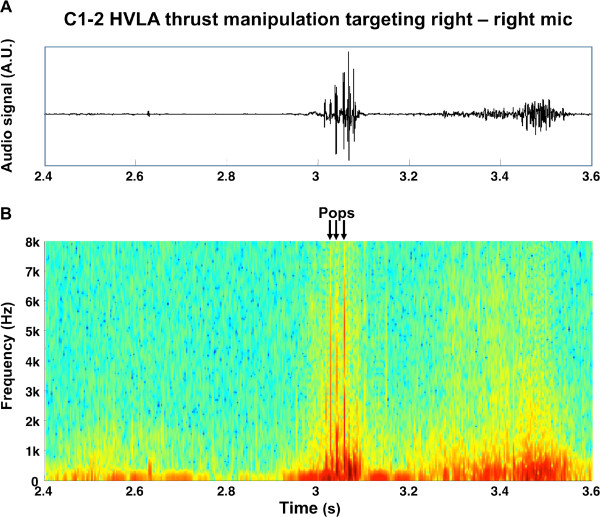
**Raw audio signal (above) detected during a C1-2 HVLA thrust manipulation and the corresponding spectrogram (below).** The colors are normalized to the maximum value of the spectrogram in that time epoch (red high amplitude, blue-green low amplitude). The three identified pops are visible as red vertical lines and are indicated with arrows.

Sound waves resulting from the upper cervical HVLA thrust manipulations were displayed in graphical format (Figure 3). Each subject had one right and left graph corresponding with each thrust procedure (i.e. four graphs in total for each subject). Descriptive statistics, including frequency counts for categorical variables and measures of central tendency and dispersion for continuous variables were calculated to summarize the data. Means and standard deviations were calculated to summarize the average number of pops, the duration of upper cervical thrust manipulation, and the duration of a single cavitation. The primary aim, to determine which side of the spine cavitates during C1-2 HVLA thrust manipulation, was examined using a Chi-square test. The probability for unilateral or bilateral cavitation events was calculated using the binomial test assuming an expected probability of 50% (i.e. a reference proportion of 0.5). Data analysis was performed using SPSS 20.0.

### Data extraction

**Figure 4:**
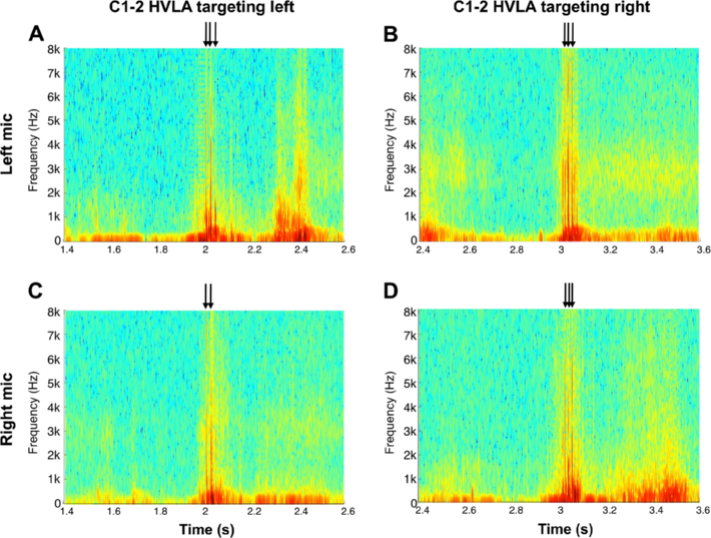
Four spectrograms from a single subject corresponding to the audio signals from two microphones over the right and left transverse processes of C1 during two separate C1-2 HVLA thrust manipulations (one targeting the left and the other targeting the right atlanto-axial articulation).

Short-Time Fourier Transformation (STFT) was used to process the sound signals and obtain spectrograms for each thrust manipulation. A spectrogram is a 2-dimensional graphical representation with time on the x-axis, frequency on the y-axis, and color as a third dimension to express the amplitude, or power of the sound (Figure 3). Each subject had one right and one left spectrogram corresponding with each thrust manipulation—i.e. four spectrograms in total per subject (Figure 4). For each audio recording, the spectrograms were computed using STFT in order to evaluate the sinusoidal frequency content of each signal over time. The frequency scale was set between 1 Hz and 2 kHz with a resolution of 1 Hz. The epoch length was set to 5 ms with 1 ms overlap between subsequent epochs.

### Process for determining the side of cavitation

For each burst of energy in the spectrogram we computed the amplitude as the average rectified value (ARV) of the signal in an epoch of 20 ms centered on the instant of maximum energy of the spectrogram. When simultaneous bursts were recorded from the left and right microphones, the side with the larger amplitude (ARV) was considered the side of the cavitation.

### Process for counting the number of cavitations

The spectrograms were visually inspected in order to identify instantaneous bursts of energy that corresponded to cavitations (Figures 3 and 4). The total number of cavitations during each manipulation was the sum of the number energy bursts identified in the left and right microphone recordings. In the event of simultaneous bursts of energy (i.e. to the 1/10,000^th^ of a second) to the left and right side, only one cavitation was counted. In other words, sound waves that arrived to the right and left microphones at exactly the same time (i.e. within 1/10,000^th^ of a second) were assumed to originate from a single cavitation.

### Process for calculating the duration of a single pop

**Figure 5:**
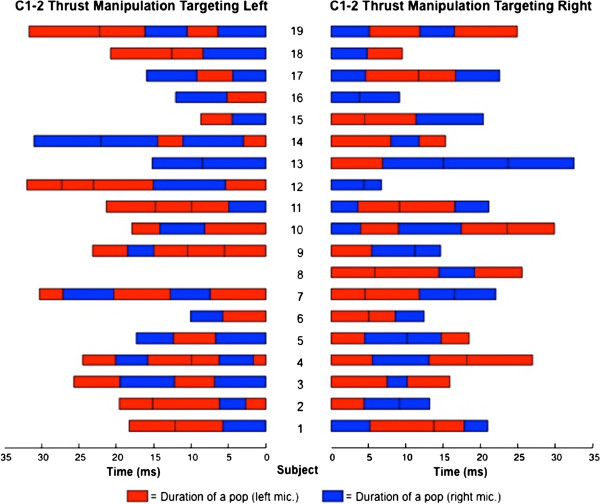
For each of the 19 subjects, the side and the duration in milliseconds for each of the 132 popping sounds during 37 separate HVLA thrust manipulations targeting the right or left C1-2 articulation.

The time interval that included 95% of the sound wave energy was used to calculate the duration of individual popping sounds that were detected during the 37 upper cervical thrust manipulation procedures (Figure 5). The signal epoch that included a pop was defined as a 20 ms epoch centered on the instant of maximum energy of the spectrogram relative to that popping sound. The total energy of the epoch was computed as the ARV of the 20 ms signal epoch and the process was iteratively applied reducing the epoch length progressively until the ARV was 95% of the original value.

### Process for calculating the duration of the thrust manipulation

**Figure 6:**
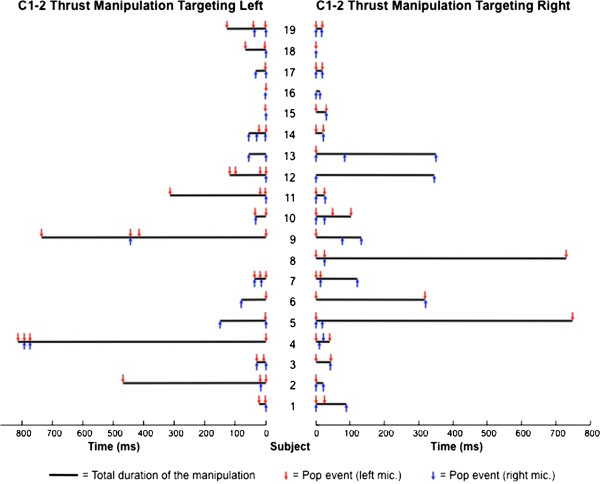
For each of the 19 subjects, the side and time point of occurrence in milliseconds for 132 popping sounds (red and blue arrows) and the total duration in milliseconds for 37 C1-2 rotatory HVLA thrust manipulations (black horizontal bars) following spectrogram analysis are depicted.

The time between the first and last popping sound of each thrust manipulation was considered the duration of the thrusting procedure (Figure 6). However, although we did not measure the actual forces against time, the duration of the thrust manipulation used in our study likely does not include the time from when the force beyond the preload first began to be applied or the entire interval from when the peak forces dropped back to zero [28,36,37]. In case only one popping sound was observed, the duration of the thrust manipulation was considered equal to the duration of that popping sound.

## Results

Of the 132 total cavitations, 72 occurred ipsilateral and 60 occurred contralateral to the targeted C1-2 articulation; that is, cavitation was no more likely to occur on the ipsilateral than the contralateral side (Pearson Chi-square = 1.100; P = 0.294) following right or left rotatory C1-2 HVLA thrust manipulation (Figures 5 and 6). More specifically, when targeting the left C1-2 articulation, bilateral popping sounds were detected in 17 (94.4%) of the 18 upper cervical rotatory HVLA thrust manipulations, whereas unilateral popping sounds were detected in just 1 (5.6%) of the thrust manipulations. Likewise, when targeting the right C1-2 articulation, bilateral popping sounds were detected in 17 (89.5%) of the 19 upper cervical rotatory HVLA thrust manipulations, whereas unilateral popping sounds were detected in just 2 (10.5%) of the 19 thrust manipulations (Figure 5).

Bilateral popping sounds were detected in 34 (91.9%) of the 37 upper cervical rotatory HVLA thrust manipulations and unilateral popping sounds were detected in just 3 (8.1%) of the 37 thrust manipulations; that is, cavitation was significantly (binomial Test, P < 0.001) more likely to occur bilaterally than just unilaterally (Figures 5 and 6). Moreover, during upper cervical rotatory HVLA thrust manipulation targeting the right or left atlanto-axial joint, the resulting popping or cracking sounds were 11.3 times more likely to occur bilaterally than just unilaterally.

All 37 upper cervical HVLA thrust manipulations resulted in two or more audible joint popping sounds. One hundred thirty-two popping sounds were detected following 37 upper cervical thrust manipulations giving a mean of 3.57 (95% CI: 3.19, 3.94) distinct pops per C1-2 HVLA thrust manipulation procedure. Two distinct popping sounds were produced in 7 (18.9%) of the manipulations, whereas 11 (29.7%), 12 (32.4%), 5 (13.5%), and 2 (5.4%) manipulations produced 3, 4, 5, and 6 distinct popping sounds, respectively. Nineteen subjects received 37 manipulations (two each, with the exception of one subject whose data was not retrievable after one of the manipulations); therefore, the mean number of pops per subject after right and left C1-2 HVLA thrust manipulation (two separate thrust procedures) was 6.95 (95% CI: 6.11, 7.79) with a range of 3 to 10 pops.

The mean duration of a single pop was 5.66 ms (95% CI: 5.36, 5.96) and the mean duration of a single upper cervical rotatory HVLA thrust manipulation was 96.95 ms (95% CI: 57.20, 136.71).

## Discussion

### Side of the cavitation

To our knowledge, this the first study to identify the side of joint cavitation during upper cervical HVLA thrust manipulation. Our results indicate that cavitation was no more likely to occur on the ipsilateral than the contralateral side following right or left rotatory C1-2 HVLA thrust manipulation. In addition, bilateral popping sounds were detected in 34 (91.9%) of the 37 upper cervical rotatory HVLA thrust manipulations, while unilateral popping sounds were detected in just 3 (8.1%) of the 37 thrust manipulations. Resulting popping sounds were 11.3 times more likely to be a bilateral “event” than just a unilateral “event”.

It is difficult to directly compare the results of our study with the two previous studies [2,8] on this topic because our study is the first to investigate the side of cavitation during upper cervical C1-2 HVLA thrust manipulation and to use spectrogram analysis of sound waves. Nevertheless, it is noteworthy that both Reggars and Pollard [8] and Bolton et al. [2] reported that the popping was significantly more likely to occur on the contralateral side to the applicator contact, a finding in direct contrast to the present study. However, this was during “lateral to medial and rotatory” [8] or “rotatory” [2] manipulations to the C3-4 articulation, not rotatory HVLA thrust manipulations targeting the C1-2 segment that were used in our study.

In addition, the upper cervical thrust technique used in our study cannot be considered synonymous with the mid-cervical thrust technique in the other two studies; [2,8] that is, in addition to contralateral rotation and side-bending levers, we also used contralateral translation and PA shift, two accessory motions, that were likely not employed by the two previous studies [2,8]—perhaps this, in part, explains why our findings were 92% bilateral “events” rather than 94% and 80% just contralateral “events” as reported by Reggars and Pollard [8] (C3-4 “lateral to medial and rotatory” thrust) and Bolton et al. [2] (C3-4 “rotation manipulation”), respectively. In addition, 15 of the 50 asymptomatic subjects in the Reggars and Pollard [8] study had a “history of neck trauma” which could have theoretically altered the arthrokinematics of the cervical spine [38,39].

Our study also mounted microphones directly over the target vertebra (i.e. the lateral aspect of the transverse process of C1), while both Bolton et al. [2] and Reggars and Pollard [8] mounted microphones over the articular pillar and transverse process, respectively, of the C2 vertebra when the target was the C3-4 articulation in each of those studies. In addition, Bolton et al. [2] used a considerably lower sampling frequency of 2000 Hz (compared to 44,100 Hz in our study). As a result, they were only able to analyze signal amplitude in determining the side of the pop. That is, rather than analyzing time intervals and signal amplitudes between bilateral microphones (to 1/10,000^th^ of a second as we did) to determine side of cavitation sound, Bolton et al. [2] used signal magnitude as the sole indicator for allocating the side of cavitation. More specifically, Bolton et al. [2] assumed that the side with the larger amplitude sound wave was the side of “initial cavitation” and did not report if single or multiple cavitations occurred. Unless single cavitations occurred during all cervical manipulations, which is unlikely given the existing literature, [1,4,8-10] the possibility remains that the “initial cavitation” occurred on one side and additional cavitations followed that were ipsilateral and/or contralateral. Therefore the results of Bolton et al. [2] should be viewed cautiously.

Of the 132 total cavitations identified in our study, 72 occurred ipsilateral and 60 occurred contralateral to the targeted C1-2 articulation; that is, cavitation was no more likely to occur on the ipsilateral than the contralateral side. Therefore, for practitioners that wish to target a specific dysfunctional vertebral segment of the upper cervical spine, as has been traditionally taught in manual therapy, [3,6,24,26] and based on the results of our study, it may be appropriate to perform the C1-2 HVLA thrust manipulation from both sides, [5,25,40] to maximize the likelihood that the target articulation was indeed “cracked” or “popped”.

### Number of pops per thrust

To our knowledge, this is the first study to investigate the number of popping sounds during HVLA thrust manipulation to the C1-2 articulation. We identified 132 popping sounds following 37 upper cervical thrust manipulations resulting in a mean of 3.57 distinct pops and a range of 1 to 7 pops per C1-2 HVLA thrust manipulation. Similarly, after 50 manipulations in 50 subjects, Reggars [10] reported 123 individual joint cracks resulting in a mean of 2.46 pops and a range of 1–5 pops per C3-4 HVLA thrust manipulation. That is, Reggars [10] found the majority of subjects (64%) produced 2–3 distinct popping sounds, whereas, the present study found that the majority of subjects produced 3–4 popping sounds. Similarly, Reggars and Pollard [8] reported 116 individual joint cracks in 50 subjects following 50 thrust manipulations targeting C3-4 (giving a mean of 2.32 cracks per manipulation) with only 24% of subjects producing a single joint crack and 64% producing 2 or 3 distinct joint cracks (range of 1–5 pops per manipulation).

Although in a different spinal region, Ross et al. [9] found 1–6 audible popping sounds per thoracic or lumbar HVLA thrust manipulation. In 8 of 30 of subjects, Cramer et al. [4] further found 2 or more popping sounds per lumbar HVLA thrust manipulation. The traditional expectation of achieving just one single pop per HVLA thrust manipulation in the cervical, thoracic, or lumbopelvic regions is therefore not supported by the existing literature; [1,4,8-10] and “one pop” should no longer be taught as the “goal” or “expectation” in conventional manual therapy training programs. Nevertheless, to date, no study has investigated the clinical significance (i.e. its relationship to pain and disability) of the popping sounds following cervical HVLA thrust manipulation in patients with neck pain.

Whether the 3–4 popping sounds that we found in our study emanated from the same joint, adjacent ipsilateral or contralateral facet or uncovertebral joints, or even extra-articular soft-tissues has yet to be determined. A recent study [4] reported detecting “multiple cavitations from individual zygapophyseal joints” following lumbar HVLA thrust manipulation in 8 of 40 healthy subjects; however, the internal validity of this study must be carefully considered as participants received “2 thrusts” to the same region and only two pops were recorded in 7 of the 8 subjects. Moreover, the origin of the vibrations detected by the accelerometers taped to the spinous processes during HVLA thrust manipulations remains to be elucidated. It is only theorized to be an intra-articular phase change of carbon dioxide and actual “cavities” in zygapophyseal joints have yet to be visualized during or immediately following HVLA thrust manipulation of any spinal region [11,15,41]. That is, the claim by Cramer et al. [4] that they recorded “multiple cavitations from individual zygapophyseal joints” is not supported by the methods of the study because the vibrations recorded by the accelerometer may just as likely be from extra-articular soft-tissue events [11]. Unlike the MCP joint where post manipulation increases in joint space and decreases in joint density have been observed, [12,14] Cascioli et al. [11] found no evidence of gas in the joint space (i.e. no “cavities” or vacuum phenomenon) and no evidence of increased zygapophyseal joint width, using CT scans and plain film images, immediately following cervical HVLA thrust manipulation.

Notably, each cervical vertebra is involved in 4 facet joints, and each vertebra at C2 and below also has 4 uncovertebral joints; thus, it may be theoretically possible that any one or combination of these joints may be cavitated during a thrust manipulation to the cervical spine. Certainly, it seems unlikely that the 3–4 popping sounds we found in most subjects in our study emanated from a single facet joint.

### Duration of an individual pop

We found the mean duration of a single pop to be 5.66 ms (95% CI: 5.36, 5.96). This value is very similar to the 4 ms duration reported by Reggars and Pollard [8] for the “average length of joint crack sounds”. We are aware that this value is considerably smaller than the values reported by Sandoz et al. [14] (40–60 ms) and Meal and Scott [29] (25–75 ms). However, they [14,29] investigated thrust manipulation to the MCP joints, not the cervical spine as we did. Although, Herzog et al. [7] reported triphasic “cavitation signals” with a mean duration of 20 ms, it is unclear whether this value represents a single popping sound or multiple popping sounds. However, in our study, we calculated the time interval that included 95% of the sound wave energy. The interval was therefore representative of the duration of 132 individual popping sounds detected during 37 upper cervical thrust manipulation procedures.

### Duration of the thrust procedure

Unlike previous studies, [7,26,27] we used the time interval between the first and last popping sound of each thrust manipulation to represent the duration of the actual thrusting procedure from onset to arrest; nevertheless, we found the mean duration of a single upper cervical rotatory HVLA thrust manipulation to be 96.95 ms (95% CI: 57.20, 136.71), a value that is consistent with Triano [26] (135 ms), Herzog et al. [7] (80–100 ms) and Ngan et al. [27] (158 ms). However, to date, our study is the first to report a duration for the HVLA thrusting procedure targeting specifically the C1-2 upper cervical articulation.

### Clinical relevance of the cavitation sounds

The cavitation sound is traditionally considered by many practitioners to be an important indicator for the successful technical delivery of an HVLA thrust manipulation [3,4,6,7,9,20,21,24,26]. However, four studies [22,23,42,43] have suggested that the audible pop following thrust manipulation is not related to clinical outcomes. While these studies [22,23,42,43] investigated the thoracic and lumbopelvic regions and not the cervical spine, anecdotal evidence suggests that there is an association between clinical outcomes and the popping sound. In fact, many clinicians [3,6,24] and research teams [4,5,19-21,40,44-46] still repeat the HVLA thrust manipulation if they do not hear or palpate popping sounds. Moreover, Evans and Lucas [47] recently provided five empirically-derived features proposed to be necessary components of a valid manipulation, one of which was “cavitation within the affected joint”. In other words, the audible popping, or the “mechanical response” that “occurs within the recipient”, should be present to satisfy the proposed manipulation criteria [47].

### Risks of upper cervical HVLA thrust manipulation

Considerable attention has been given to the potential risks associated with HVLA thrust manipulation procedures in the cervical region [30,31,34,48-50]. Although beyond the scope of the current article, the most recent study by Cassidy et al. [49] provides robust evidence for the risk of vertebrobasilar artery (VBA) stroke and cervical HVLA thrust manipulation. Contrary to traditionally held views, [51,52] Cassidy et al. [49] found no greater risk of VBA stroke associated with cervical HVLA thrust manipulation than general, primary medical physician care. Moreover, a recent systematic review [48] concluded there is no strong evidence linking the occurrence of serious adverse events with the use of cervical manipulation or mobilization in adults with neck pain.

The two largest randomized controlled trials [53,54] within the past 10 years comparing the effectiveness of cervical HVLA thrust manipulation with cervical non-thrust mobilization did not report the specific vertebral motion segment targeted with the cervical HVLA thrust manipulation procedure. Therefore, it is unknown whether patients with acute or chronic neck pain in these studies received upper, middle or lower cervical HVLA thrust manipulation [53,54]. Notably, there were no serious neurovascular adverse events reported by the participants in either of the trials, [53,54] and both trials reported no statistically significant difference in the incidence of minor adverse events between the cervical HVLA thrust manipulation and cervical non-thrust mobilization groups. Therefore, to date, there is no strong empirical evidence to support the notion that upper cervical HVLA thrust manipulation carries any greater risk of injury than middle or lower cervical HVLA thrust manipulation, or that non-thrust mobilization to any region of the cervical spine carries any less risk than HVLA thrust manipulation to the same region [31,48-50].

### Limitations

The results of this study may not be generalizable to the middle and lower cervical spine because of the differences in the morphology and arthrokinematics of the zygapophyseal joints in these regions and the upper cervical spine. Furthermore, the results of our study cannot be generalized to upper cervical manipulation techniques that use different combinations of primary and secondary, physiologic or accessory component levers. One further limitation of this study is that only one practitioner administered all of the upper cervical thrust manipulations; hence, it can’t be assumed that the individual and subtle nuances to technique delivery adopted with time and experience would be identical in other practitioners administering the same procedure. Future research should determine the vertebral level (or levels) at which the popping sounds are emanating from and investigate the clinical significance of the cavitation phenomenon following upper cervical HVLA thrust manipulation in patients with mechanical neck pain, cervicogenic headache, whiplash associated disorder, or other such subgroups. In addition, future trials should investigate whether a relationship exists between the number of cavitations and the degree of change in the clinical outcomes of pain and disability in these subgroups of patients.

## Conclusion

Cavitation was significantly more likely to occur bilaterally than unilaterally during upper cervical HVLA thrust manipulation; that is, the popping sounds associated with C1-2 manipulation were 11 times more likely to occur bilaterally than just unilaterally. Most subjects produced 3–4 pops during a single rotatory HVLA thrust manipulation targeting the right or left C1-2 articulation; therefore, practitioners of spinal manipulative therapy should expect multiple popping sounds when performing upper cervical thrust manipulation to the atlanto-axial joint. Furthermore, the traditional manual therapy approach of targeting a single ipsilateral or contralateral facet joint in the upper cervical spine may not be realistic.

Whether the multiple popping sounds found in this study emanated from the same joint, adjacent ipsilateral or contralateral facet or uncovertebral joints, or even extra-articular soft-tissues remains to be elucidated.

## Competing interests

The authors declare that they have no competing interests.

## Authors’ contributions

JD participated in the conception, design, data acquisition, statistical analyses, and drafting of the manuscript. FM participated in the design, initial selection of subjects, data interpretation and revision of the manuscript. MB participated in the conception, design, data collection, statistical analyses, and revision of the manuscript. DL participated in the design, data collection, drafting of the manuscript, and revision of the manuscript. CC participated in data extraction, statistical analyses, interpretation of data, and revision of the manuscript. RB was involved in the interpretation of data, drafting of the manuscript and critical revision of the manuscript for important intellectual content. All authors read and approved the final manuscript.

## Pre-publication history

The pre-publication history for this paper can be accessed here:

http://www.biomedcentral.com/1471-2474/14/24/prepub
